# NMT1 inhibition modulates breast cancer progression through stress-triggered JNK pathway

**DOI:** 10.1038/s41419-018-1201-x

**Published:** 2018-11-16

**Authors:** Lu Deng, Xinlei Gao, Bingjie Liu, Xueyan He, Jiahui Xu, Jiankun Qiang, Qingfa Wu, Suling Liu

**Affiliations:** 1 Fudan University Shanghai Cancer Center and Institutes of Biomedical Sciences; Shanghai Medical College; Key Laboratory of Breast Cancer in Shanghai; Innovation Center for Cell Signaling Network; Cancer Institutes, Fudan University, 200032 Shanghai, China; 2 School of Life Science, The CAS Key Laboratory of Innate Immunity and Chronic Disease, University of Science and Technology of China, 230027 Hefei, Anhui China

## Abstract

Myristoylation is one of key post-translational modifications that involved in signal transduction, cellular transformation and tumorigenesis. Increasing evidence demonstrates that targeting myristoylation might provide a new strategy for eliminating cancers. However, the underlying mechanisms are still yielded unclear. In this study, we demonstrated that genetic inhibition of N-myristoyltransferase NMT1 suppressed initiation, proliferation and invasion of breast cancer cells either in vitro or in vivo. We identified ROS could negatively regulate NMT1 expression and NMT1 knockdown conversely promoted oxidative stress, which formed a feedback loop. Furthermore, inhibition of NMT1 caused degraded proteins increase and ER stress, which cross-talked with mitochondria to produce more ROS. And both of oxidative stress and ER stress could activate JNK pathway, leading to autophagy which abrogated breast cancer progression especially triple-negative breast cancer (TNBC). These studies provide a preclinical proof of concept for targeting NMT1 as a strategy to treat breast cancer.

## Introduction

Breast cancer is one of the leading causes for mortality of women around the world. Genomic studies have identified five major breast cancer intrinsic subtypes: luminal A, Luminal B, HER2-enriched, basal-like, and claudin-low, that show significant differences in incidence, survival, and response to therapies^1,2^. Unlike other subtypes, basal-like and claudin-low breast cancers still lack effective ways of treatment due to absence of approved hormone, targeted therapeutic options and frequently poor response to standard chemotherapies^3^. Previous reports demonstrated that basal-like, especially claudin-low subtype, is enriched for breast tumor initiating cells (BTIC) features^4–6^. Our previous studies have shown that BTIC with the enzyme aldehyde dehydrogenase (ALDH) activity (ALDH-positive) are enriched for tumor-initiating characteristics^7^. Therapeutic target on ALDH positive population might provide insights to treat triple-negative breast cancers.

NMT1 is an enzyme for catalyzing myristoylation of over 100 proteins in human cells^8^. Myristoylation is a co-translational and post-translational modification in eukaryotes, which transfers myristate to the N-terminal glycine of substrate proteins by NMT1 and NMT2^9^. Previous reports have shown that NMT1 was related to lots of carcinoma due to the substrates of which are involved in a wide variety of signal cascades, cellular transformations and oncogenesis^8,10^. Recent study has demonstrated that Src needs NMT1 to help promote prostate cancer progression^11^. In breast cancer, utilizing a NMT inhibitor to block the whole myristoylation causes ER stress and apoptosis^12^. However, there are few studies have specifically examined the role of prolonged inhibition of NMT1 on cancer. And the mechanisms of what regulating NMT1 expression is still not known yet.

In this study, we explored the role and mechanisms of NMT1 in regulating breast cancer initiation, progression and metastasis. We specifically focused our research on triple-negative breast cancer (TNBC) and found that genetic inhibition of NMT1 triggered both ER stress and oxidative stress, and therefore stimulating the JNK pathway to inhibit breast cancer progression. These data provide an innovative aspect for future studies to decipher the action mode of NMT inhibition and the validation of NMT1 as a therapeutic target for clinically use in breast cancer.

## Materials and methods

### Cell culture and reagents

The human breast cancer cell line SUM149 was got from Asterland Bioscience, which was cultured in F12 medium with 5% fatal bovine serum (FBS) (Thermo Fisher) and 1% streptomycin/penicillin (Beyotime), 1 mg/ml hydrocortisone, and 5 mg/ml insulin. MDA-MB-231, HCC1937, and T47D were obtained from ATCC and were cultured according to ATCC recommendations. These cells are maintained in a 37 °C incubator with 5% carbon dioxide (CO2). Sodium phenylbutyrate (4-PBA), Brefeldin A (BFA) and SP600125 were purchased from MCE and dissolved in DMSO. N-acetyl cysteine (NAC) (Beyotime) was dissolved in distilled sterile water.

### Human transcriptome array analysis and miR-100 target gene identification

Gene expression profiles were analyzed using Affymetrix Human Transcriptome Array 2.0 (HTA 2.0) microarray data of miR-100 over-expressing SUM159 and MDA-MB-231 cell lines and the control cell lines. The raw data was normalized and compared using the Expression Console and Transcriptome Analysis Console software provided by Affymetrix Corporation. Differentially expressed genes between miR-100 over-expressing cells and the control cells were identified with fold change >1.5. MiR-100 target genes were collected from three microRNA databases, namely microRNA.org (http://www.microrna.org)^13^, TargetScan (www.targetscan.org)^14^ and PITA (https://genie.weizmann.ac.il)^15^. MiR-100 target genes down-regulated by at least 1.5 folds in the miR-100 over-expressing SUM159 or MDA-MB-231 cell lines were retrieved for further investigation.

### Plasmid constructs and lentiviral infection

PTRIPZ-miR100 lentivral vector was used to overexpress miR100 as previously described^16^. Effective ShRNA sequences of NMT1, PERK, IRE1A, and ATF6 were cloned into PLKO.1 plasmid from Sigma-Aldrich. The full-length human NMT1 ORF was generated and cloned into the lentiviral vector pSIN with a FLAG tag (Addgene). Virus packaging and cell transfection were performed as described previously. ShRNA sequences were provided in Table S1.

### Flow cytometry

For the ALDEFLUOR assay (StemCell), dissociated single cells were suspended in assay buffer contain ALDEFLUOR substrate and incubated with or without DEAB. Analysis of tumor cell suspensions from xenograft tumors were performed as previous report. Briefly, PE-conjugated anti-mouse lineage antibodies (CD45 (BD), CD31 (BD), CD140b (BD), CD235a (BD), and H2KD (Biolegend)) were used for gating out non-breast cancer cells. For cell cycle analysis, cells were fixed with 70% alcohol at 4 °C overnight and stained with propidium iodide (Sigma-Aldrich) in the presence of 1% RNAase A (Takara) at 37 °C for 30 min prior to analysis. For apoptosis analysis, cells were stained with propidium iodide (Sigma-Aldrich) and Annexin V (BD), according to the manufacturer’s instructions. DCFH-DA (Sigma-Aldrich) was added to the cells without FBS for detecting ROS production. CytoFLEX (Beckman Coulter) was used for detection and data acquisition and analysis were performed in CytoExpert software. A MoFlo Astrios instrument (Beckman Coulter) was used for sorting cells.

### Mammosphere formation assay

200 tumor cells were cultured with MammoCult Human Medium Kit (StemCell) supplemented with 4 µg/mL Heparin (StemCell), 1 ug/mL hydrocortisone (Sigma-Aldrich) and 1% pen-strep antibiotic (Beyotime) in 96-well ultra-low attachment plates (Corning) for about two weeks. Fresh complete mammocult medium was added every 3 days. Sphere number and size then observed and photographed for further statistical analysis.

### Total RNA isolation and qRT-PCR

Total RNA was extracted using the RNAiso Plus reagent (Takara). Complementary DNA (cDNA) was prepared from 1 µg RNA using the HiScript II 1st Strand cDNA Synthesis Kit (Vazyme Biotech). QRT-PCR was carried out using AceQ qPCR SYBR Green Master Mix (Vazyme Biotech) in a real-time PCR system (7300, Applied Biosystems). There were three replicates for each gene in parallel. TATA-box binding protein (TBP) was used as a reference gene. For quantification of miR100, U6 was used as a reference gene. QRT-PCR primers were provided in Table S1.

### Western blotting

Cells were lysed in RIPA buffer (Beyotime). Protein lysates mixed with loading buffer were separated by SDS-PAGE and transferred onto PVDF membranes (Millipore). Membrane was blocked in 5% de-fat milk and incubated with primary antibody at 4 ℃ overnight and HRP-conjugated secondary antibody at room temperature for 1 h sequentially. Chemi-luminescent detection was performed using an ImageQuant LAS 4000 mini imaging system (GE) with Western HRP Substrate (Millipore). Following antibodies were used in this study: anti-NMT1 (11546-1-AP, Proteintech), anti-ACTIN (HC201, TransGen), anti-BIP (3177, CST), anti-PERK (5683, CST), anti-IRE1A (3294, CST), anti-ATF6 (24169-1-AP, Proteintech), anti-JNK (9252, CST), anti-Phospho-JNK (4668, CST), anti-Phospho-AKT (4058, CST), anti-Phospho-ERK (9101,CST), anti-Phospho-Rb (8516), anti-CyclinA2 (4656, CST), anti-CyclinB1 (12231, CST), anti-BECLIN (3495, CST), anti-ATG12 (4180, CST), anti-LC3B (18725-1-AP, Proteintech), anti-P53 (9284, CST), anti-P21(P1484, Sigma-Aldrich), anti-Phospho-P53 (7907, CST), goat anti-mouse IgG-HRP (sc-2005, Santa Cruz) and goat anti-rabbit IgG-HRP (sc-2004, Santa Cruz).

### Immunoprecipitation and functional enrichment analysis

Cells were collected and lysed in EBC buffer [PH 7.5] (50mMTris, 120 mM NaCl, 0.5% NP40) containing PMSF (Beyotime) and Phosphatase inhibitor (Roche). After centrifugation, supernatant was incubated with flag-beads (Sigma-Aldrich) at 4 °C overnight. The bound proteins were washed with NETN buffer [PH 8.0] (20 mMTris, 100 mM NaCl, 0.5% NP40, 1 mM EDTA) with PMSF and Phosphatase inhibitor, four times and sent to mass spectrometric analysis.

Gene Ontology (GO) enrichment analysis for gene lists from mass spectrometric analysis in the SUM149 NMT1 overexpressing cells was conducted using PANTHER Overrepresentation Test^17^, enriched biological process terms with False Discovery Rate (FDR) <0.05 were regarded as significant, and the redundant enriched terms were removed with REVIGO^18^. The top 10 significantly enriched GO terms were plotted using custom R scripts for SUM149 cells. Heatmap of gene expression profiles was produced by the R package pheatmap (https://cran.r-project.org/web/packages/), with log10 transformed gene LFQ intensity. Protein analyzed by mass spectrometric was listed in Table S2.

### Antibody array

Cells were collected and used for human Phospho-Kinase Array (R&D Systems) test following the manufacturer’s instruction. Chemi-luminescence detection was performed and signal intensity was digitalized with ImageJ software.

### Immunohistochemistry

Patient breast cancer tissues and their corresponding adjacent normal tissues were obtained from Shanghai cancer hospital affiliated with Fudan University. The sections of paraffin-embedded human tissues or xenograft tumors were dewaxed and rehydrated in xylene and graded alcohol solutions. Anti-NMT1 (1:200, Proteintech), anti-Ki67 (1:200, ZSGB-BIO) were used as primary antibodies. Lung tissues of xenograft mice were stained with hemato-xylin and eosin (ZSGB-BIO).

### MTT assay

500 Cells were seeded in 96-well plates per well and cultured for 3, 5, and 7 days. Each group was performed triplicate. For each well 20 µl MTT (5 mg/ml, Biosharp) was added and plates were incubated at 37 °C for 2 h. After removing the supernatant, 100 µl DMSO per well was added and the optical density (OD) value was measured at 490 nm with microplate reader (Elx800, BioTek).

### Colony formation assay

For plate colony formation assay, 500 cells were seeded and cultured in normal condition for 2 weeks, and fixed using 10% formaldehyde for 30 minutes. The cell colonies were stained with 0.1% crystal violet for 30 minutes. After washing, 1 ml methanol was added to each well and kept shaking for 2 h. Then the optical density (OD) value was measured at 490 nm with microplate reader (Elx800, BioTek).

### Wound healing assay

Cells were seeded in 6-well plates and grown to approximately 90% confluence. After mitomycin-C (5 ug/ml) treatment for two hours, wounds were created using a pipette tip and medium without serum was added. Wound healing within the scrape lines were then observed and photographed at 24 h. Each experiment was repeated at least three times.

### Invasion assay

Transwell chambers (Corning) placed in 24-well plate were pre-coated with Matrigel (Corning) and serum-free medium for 4 h in incubator. 40,000 cells were then seeded without serum. The indicated medium with 10% FBS was added in the bottom well. After being cultured for 36 h, cells were fixed (methanol: acetic acid = 3:1) and stained with 0.1% crystal violet, and the invaded cells were photographed for statistical analysis.

### In vivo tumorigenicity

Three- to four-week-old female nude mice were obtained from vitalriver (Beijing, China) and housed in standard animal cages under specific pathogen-free conditions in Department of Laboratory Animal Science of Fudan University. All mouse experiments were conducted in accordance with standard operating procedures accordance with the recommendations in the Guide for the Care and Use of Laboratory Animals of Fudan University, and approved by the Committee on the Ethics of Animal Experiments of Fundan University. Tumors were monitored weekly until mice were sacrificed when the diameter of tumors reached 1.5–2.0 cm. Tumor volume was calculated as Length × Width^2^/2. As for the JNK inhibitor treatment experiments, the SP600125 was first dissolved in 10% DMSO/corn oil at 10 mg/ml. The stock solution was diluted in corn oil (Solarbio) to prepare 200 μl solutions of SP600125 for each injection. The SP600125 solutions were injected intraperitoneally into nude mice (30 mg/kg/day) for two weeks. Control mice received same volume of DMSO diluted in corn oil. Each mice experiment was performed in triplicate and data were derived from at least 3 independent experiments.

### Statistical analysis

All data were presented as the mean ± standard deviation. At least three repeated individual experiments were performed for each group, except where otherwise indicated. Difference between two groups was analyzed using Student *t* test. Difference between three or more groups was analyzed by One-way/Two-way ANOVA with GraphPad Prism 6. *P* < 0.05 was considered statistically significant.

## Results

### NMT1 knockdown inhibits breast cancer initiation, growth, and metastasis in vitro or in vivo

**Fig. 1:**
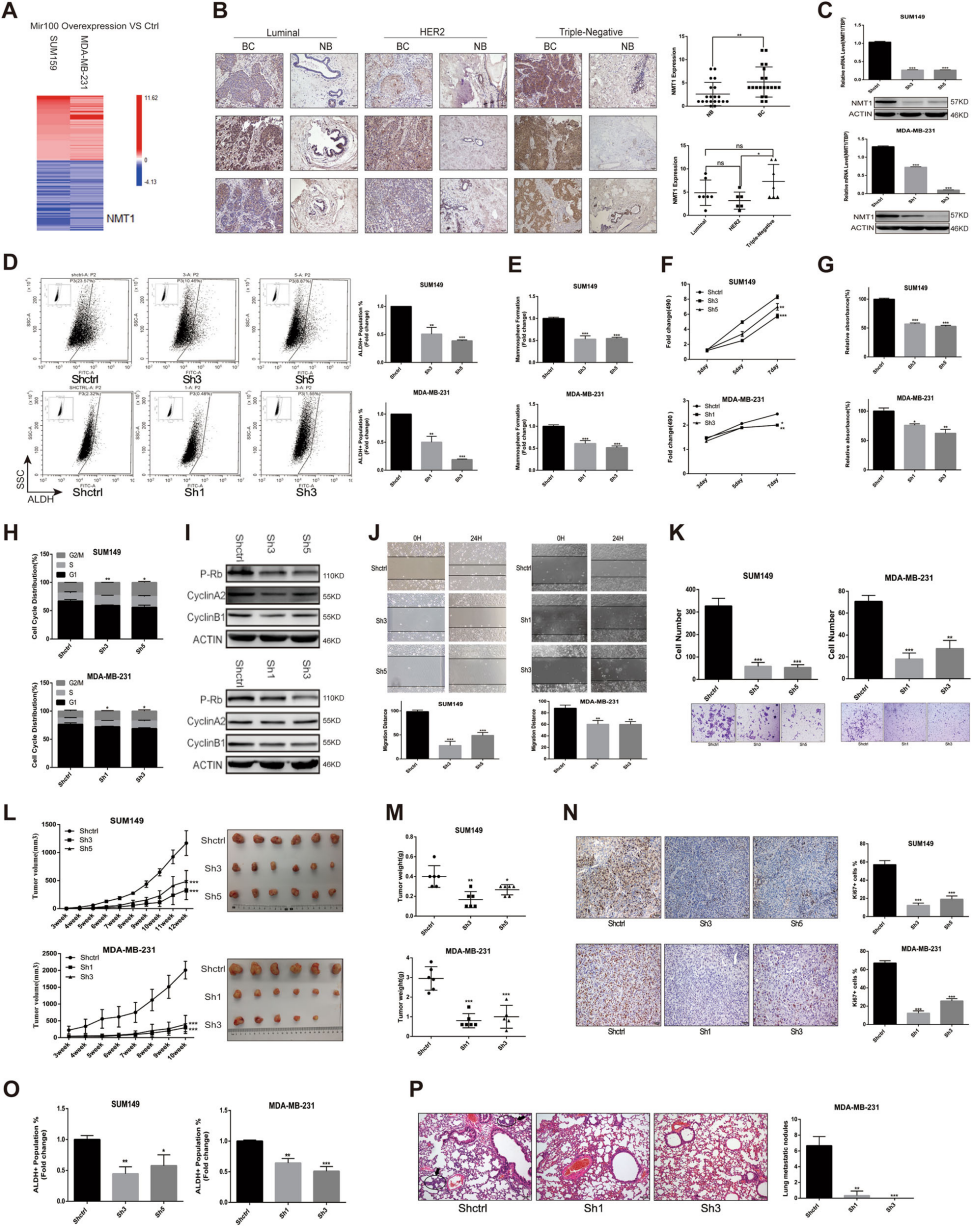
NMT1 knockdown inhibits breast cancer initiation, growth, and metastasis. **a** Heatmap from Microarray analysis representing the up- and down-regulated genes in Mir100 overexpression versus Ctrl cells both in SUM159 and MDA-MB-231. **b** NMT1 expression in clinical breast tumor tissues and adjacent noncancerous tissues were detected by IHC (left). Semi-quantitative IHC scores of NMT1 in breast tumor tissues and adjacent tissues (right). **c** NMT1 was knocked down via ShNMT1 lentiviral infection (Shctrl was the control). NMT1 expression was detected by qPCR and western blot in SUM149 and MDA-MB-231. **d** ALDEFLUOR assay of the Shctrl and ShNMT1-infected SUM149 and MDA-MB-231 cells (left). Quantification of ALDH-positive cells in these cells (right). **e** Mammosphere formation assay in Shctrl and ShNMT1-infected SUM149 and MDA-MB-231 cells. Number of mammospheres was calculated (*n* > 3). **f**, **g** MTT assay (**f**) and Colony formation assay (**g**) were used to measure cell proliferation activity as described in methods. **h** Cell cycle distribution analyzed by FACS in Shctrl and ShNMT1-infected SUM149 and MDA-MB-231 cells. **i** Cell cycle related proteins were detected by western blot in Shctrl and ShNMT1-infected SUM149 and MDA-MB-231 cells. **j**, **k** Wound healing assay (**j**) and Transwell assay (**k**) were used to measure cell migration and invasion ability as described in methods. **l** For each group, 500k SUM149 or MDA-MB-231 cells were implanted into the mammary glands of 3-week-old to 4-week-old female nude mice and tumor size was monitored weekly (left). The tumor image was shown on the right. **m** Tumor weight from **l**. **n** The representative images for Ki67 IHC staining in tumors from **l** (left) and quantification of Ki67 positive cells (right). **o** Tumors from **l** were collected and cells were isolated from each tumor. ALDH was accessed by the ALDEFLUOR assay on viable dissociated cells. **p** HE staining of lung sections from Shctrl and ShNMT-infected MDA-MB-231 tumor group and the numbers of metastatic lesions per lung section were counted. Black arrows represent metastatic nodules in lungs. Data represent the mean ± SD of 3 independent experiments where *P < 0.05, **P < 0.01 and ***P < 0.001

Our previous study has demonstrated that microRNA100 (Mir100) has a pivotal role in inhibiting breast cancer initiation and progression^16^. To identify involved genes in this process, we overexpressed Mir100 in SUM159 and MDA-MB-231 cells, and performed microarray to identify differentially expressed genes between the Ctrl and Overexpression groups (Fig. 1a). In combination with the prediction of different MicroRNA websites, we found out that NMT1 might be one of the dominant Mir100 targets. To confirm this result, we induced miR100 overexpression in MDA-MB-231 and SUM149 cell lines by utilizing pTRIPZ-miR100 plasmid (Fig. S1A). NMT1 was significantly down-regulated after miR100 overexpression (Fig. S1A). NMT1 expression was variable across different breast cell lines and did not correlate with molecular subtypes (Fig. S1B). To investigate the clinical significance of NMT1, we assessed NMT1 expression in primary breast cancer tissues and adjacent normal breast tissue of 20 patients. NMT1 level was significantly higher in breast tumor tissues compared to adjacent noncancerous tissues, and was especially increased in triple-negative subtypes of breast cancer (Fig. 1b).

To determine the effect of NMT1 expression on breast cancer, we established shRNA-mediated NMT1 knockdown cell lines in SUM149, MDA-MB-231, HCC1937 and T47D (Fig. 1c and S1C). Knockdown of NMT1 resulted in a significant decrease in the proportion of ALDH positive cells (Fig. 1d and S1D, S1E). Moreover, NMT1 knockdown decreased the mammosphere formation in these cell lines (Fig. 1e and S1F). NMT1 also promoted cell proliferation (Figs. 1f,g and S1G, S1H). And NMT1 knockdown induced proliferation inhibition was due to cell cycle arrest, where the percentage of cells in G2M phase was increased (Fig. 1h and S1I). The expression of proteins responsible for G2M transition was decreased in NMT1 knockdown breast cancer cell lines (Fig. 1i and S1J). Furthermore, migration and invasion ability of cells was dramatically inhibited by NMT1 knockdown (Figs. 1j,k and S1K, S1L).

To further verify the results in vivo, we injected NMT1 knockdown cell lines into fourth mammary glands of 3-week-old to 4-week-old female nude mice. NMT1 knockdown could significantly inhibit tumor growth (Fig. 1l,m). And NMT1 knockdown in tumors was confirmed by immunohistochemistry and western blot (Fig. S1M, S1N). Consistent with retarded tumor growth, the proportion of Ki67-positive cells was significantly lower in the NMT1 knockdown group (Fig. 1n). Aldefluor assay of the cells from tumors digestion showed that NMT1 knockdown dramatically decreased ALDH positive cells in vivo (Fig. 1o). Hematoxylin-eosin (HE) staining of lung sections showed there were fewer metastatic nodules in the NMT1 knockdown group compared to Shctrl group in MDA-MB-231 xenografts (Fig. 1p). Together, these results suggested knocking down NMT1 inhibited breast cancer progression and metastasis both in vitro and in vivo.

### NMT1 knockdown triggers ER stress in breast cancer

**Fig. 2:**
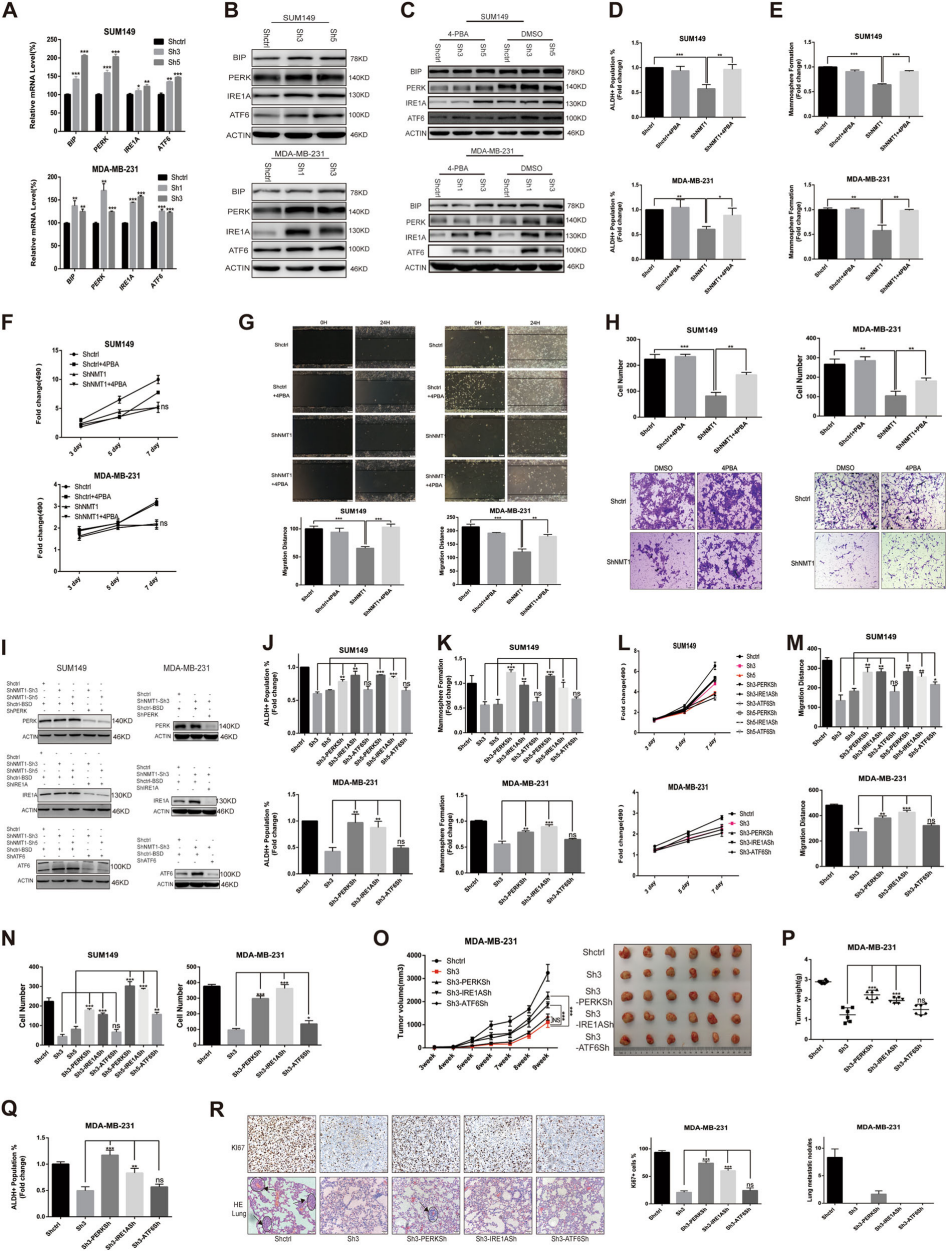
ER stress is induced by NMT1 knockdown. **a** ER stress related gene expression was determined by qRT-PCR in Shctrl and ShNMT1-infected SUM149 and MDA-MB-231 cells. **b** ER stress related proteins expression was detected by western blot. **c** Shctrl and ShNMT1-infected SUM149 and MDA-MB-231 cells were treated with 4-PBA (2uM) or same volume of DMSO for 48 h. ER stress markers were then determined by Western blot. **d** Quantification of ALDH-positive cells in 4-PBA or DMSO treated cells. **e** Quantification of Mammosphere formation in 4-PBA or DMSO treated cells. **f** MTT assay was used to measure cell proliferation activity as described in methods. **g**, **h** Wound healing assay (**g**) and Transwell assay (**h**) were used to measure cell migration and invasion ability as described in methods. **i** ER stress related genes were knocked down via lentiviral infection in Shctrl and ShNMT1-infected SUM149 and MDA-MB-231 cells. The expression of PERK, IRE1A and ATF6 was detected by western blot. **j** Quantification of ALDH-positive cells in **h**. **k** Quantification of Mammosphere formation from the cells in **h**. **l** MTT assay was used to measure the proliferation of cells in **h**. **m**, **n** Wound healing assay (**m**) and Transwell assay (**n**) were used to measure migration and invasion ability of the cells in **h** as described in methods. **o** For each group, 1 million of MDA-MB-231 cells were implanted into the mammary glands of 3-week-old to 4-week-old female nude mice and tumor size was monitored weekly (left). The tumor image was shown on the right. **p** Tumor weight from **o**. **q** Tumors from **o** were collected and cells were isolated from each tumor. ALDH was accessed by the ALDEFLUOR assay on viable dissociated cells. **r** The representative images for Ki67 IHC staining of tumors and HE staining of lung sections from **o** (left). Black arrows represent metastatic nodules in lungs. Ki67 positive cells and the numbers of metastatic lesions per lung section were counted (right).Data represent the mean ± SD of 3 independent experiments where *P < 0.05, **P < 0.01 and ***P < 0.001

Next, we searched for the possible mechanisms of how NMT1 regulating breast cancer growth and metastasis. Inhibition of NMT using chemical compound could induce ER stress in cancer cells^12^. Since this pharmacological inhibition was not specific to NMT1 and not lasted very long, we ought to elucidate whether persistent inhibition of NMT1 by shRNA would also lead to ER stress. ER chaperone BIP and ER sensors (PERK, IRE1A, and ATF6) were upregulated in NMT1 knockdown breast cancer cell lines (Figs. 2a,b and S2A, S2B), suggesting shRNA mediated NMT1 knockdown actually cause ER stress.

To investigate what role NMT1 knockdown induced-ER stress has played in regulation of breast cancer progression, we implied pharmacological and genetic inhibition of ER stress in NMT1 knockdown triple-negative breast cancer cells (SUM149, MDA-MB-231, and HCC1937). 4-phenylbutyrate (4-PBA) is a commonly used compound which could interact with unfolded or misfolded proteins to alleviate ER stress. Treatment of breast cancer cells with 4-PBA significantly reduced ER stress both in Shctrl and NMT1 knockdown groups compared to DMSO group (Fig. 2c and S2C). 4-PBA administration partially but significantly increased the NMT1 knockdown caused decrease of ALDH-positive cell population and mammosphere formation ability (Figs. 2d,e and S2D. S2E). Moreover, the decreased ability of migration and invasion of breast cancer via NMT1 knockdown was remarkably inhibited by administration of 4-PBA (Figs. 2g,h and S2G, S2H). But 4-PBA administration didn’t rescue the effect of NMT1 knockdown on proliferation (Fig. 2f and S2F). And we individually established PERK, IRE1A, and ATF6 knockdown cell lines in NMT1 knockdown breast cancer cells (Fig. 2i and S2I). Knocking down either PERK or IRE1A partially abrogated NMT1 knockdown-mediated decrease of ALDH-positive cell population and mammosphere formation (Figs. 2j,k and S2J, S2K), and partially abrogated NMT1 knockdown-mediated inhibition of migration and invasion in breast cancer cells (Figs. 2m,N and S2M, S2N). However, IRE1A or ATF6 knockdown had no effect on proliferation in NMT1 knockdown cells, and inhibition of PERK could improve a little ability of proliferation (Fig. 2l and S2L). We next determined whether ER stress had an impact on NMT1. Either ER stress inhibition via 4-PBA or activation via ER stress agonist called Brefeldin A (BFA) couldn’t affect NMT1 protein level in SUM149 (Fig. S2O, S2P), which means ER stress couldn’t inversely affect NMT1 expression.

To further investigate the role of ER stress in NMT1 knockdown cells in vivo, we injected MDA-MB-231 Shctrl, ShNMT1(NMT1 knockdown), ShNMT1-PERKSh(NMT1 and PERK double knockdown), ShNMT1-IRE1ASh (NMT1 and IRE1A double knockdown), and ShNMT1-ATF6Sh (NMT1 and ATF6 double knockdown) cells into mice. As shown in Figs. 2o,p, NMT1 knockdown inhibited tumor growth, but knockdown of PERK or IRE1A significantly promoted the growth of tumors generated from NMT1 knockdown cells, whereas ATF6 knockdown still showed no effect on tumor growth. Knockdown of three ER sensors in tumors were confirmed by western blot and IHC (Fig. 5e and S2Q). PERK or IRE1A knockdown would partially eliminate the effect of NMT1 knockdown mediated decrease of ALDH positive cell population and Ki67 positive cells in vivo (Figs. 2q,r). And HE staining of lung sections from each group showed NMT1 knockdown inhibited metastasis, which could be partially rescued by PERK knockdown (Fig. 2r). Together, these results strongly support the role of ER stress as functional downstream mediators of NMT1 knockdown in breast cancer.

### Oxidative stress regulates NMT1 expression to form a positive feedback loop

**Fig. 3:**
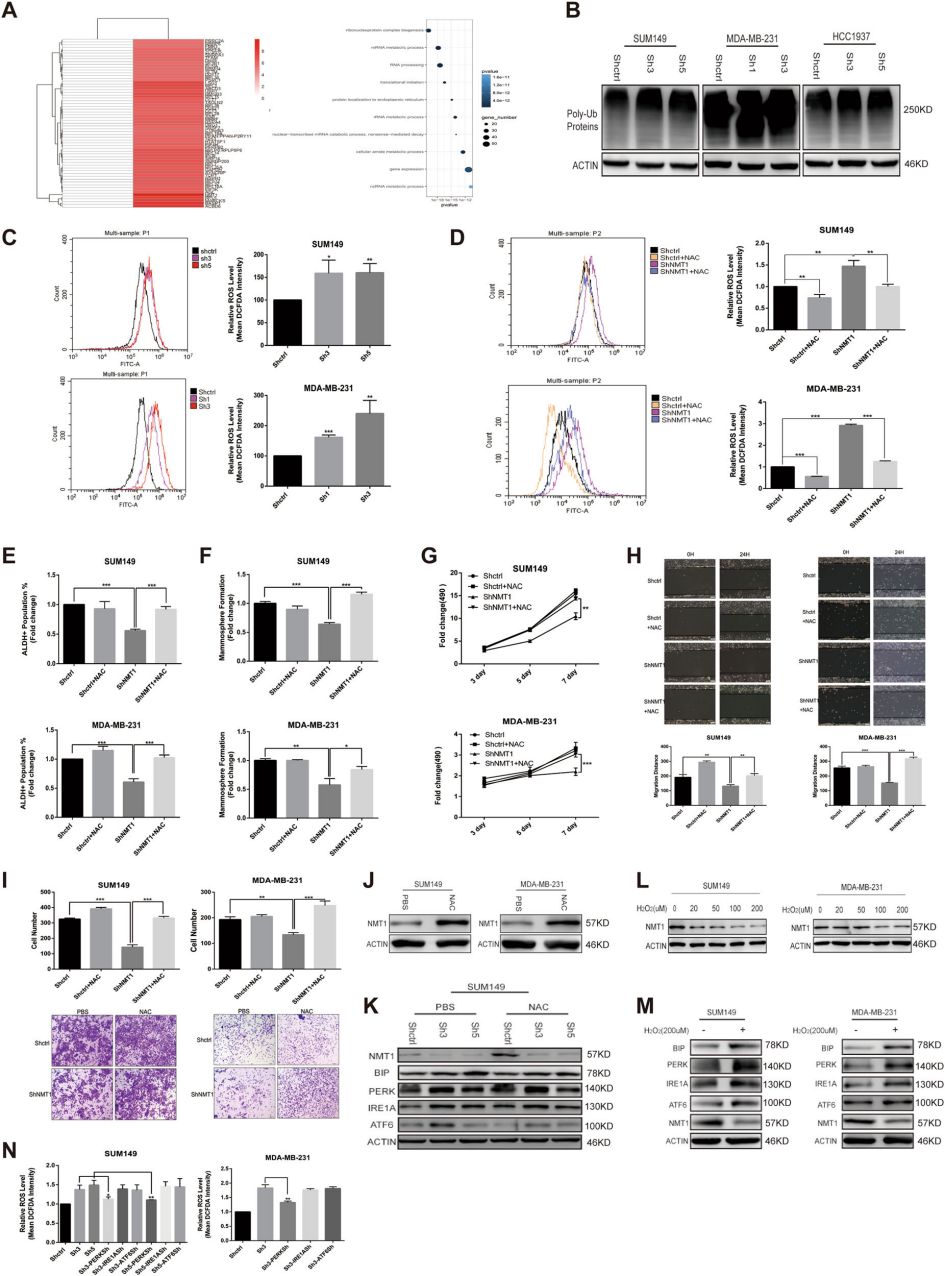
Oxidative stress regulates NMT1 expression to form a feedback loop. **a** Heatmap of the interacting proteins in NMT1 overexpressing SUM149 cells versus CTRL (left). Pathway enrichment analysis of the NMT1 interacting proteins in NMT1 overexpression SUM149 cells (right). **b** Poly-Ubiquitinated proteins were detected in Shctrl and ShNMT1-infected SUM149, MDA-MB-231, and HCC1937 cells by western blot. **c** Representative flow cytometry curves of total intracellular ROS levels (H2DCFDA) in Shctrl and ShNMT1-infected SUM149 and MDA-MB-231 cells (left). Quantification of ROS levels (right). **d** Representative flow cytometry curves of total intracellular ROS levels (H2DCFDA) in Shctrl and ShNMT1-infected SUM149 and MDA-MB-231 cells following 48 h of exposure to PBS or NAC(10 Mm) (left). Quantification of ROS levels (right). **e** Shctrl and ShNMT1-infected SUM149 and MDA-MB-231 cells were treated with NAC (10 mM) or same volume of PBS for 48 h. ALDH was accessed by the ALDEFLUOR assay on these cells and ALDH-positive cells were calculated. **f** Quantification of Mammosphere formation from the cells in **e**. **g** MTT assay was used to measure the proliferation of cells in **e**. **h–i** Wound healing assay (**h**) and Transwell assay (**i**) were used to measure migration and invasion ability of the cells in **h** as described in methods. **j** SUM149 and MDA-MB-231 cells were treated with NAC (10 mM) or same volume of PBS for 48 h. Then the expression of NMT1 was detected by western blot. **k** Shctrl and ShNMT1-infected SUM149 cells were treated with NAC (10 mM) or same volume of PBS for 48 h. Then the ER stress related proteins and NMT1 were measured by western blot. **l** SUM149 and MDA-MB-231 cells were treated with indicated concentration of H_2_O_2_ for 48 h. The expression of NMT1 was detected by western blot. **m** SUM149 and MDA-MB-231 cells were treated with 200uM H_2_O_2_ for 48 h. The expression of ER stress related proteins and NMT1 were measured by western blot. **n** ER stress related genes were knocked down via lentivirus infection in Shctrl and ShNMT1-infected SUM149 and MDA-MB-231 cells. Quantification of total intracellular ROS levels (H2DCFDA) in these cells. Data represent the mean ± SD of 3 independent experiments where *P < 0.05, **P < 0.01 and ***P < 0.001

In order to define the mechanism of how ER stress was triggered by NMT1 knockdown, we performed Co-IP and Mass Spectrum (MS) in NMT1 overexpression cells to find out NMT1 interacting proteins in SUM149. As shown in Fig. 3a, heatmap analysis displayed a quantity of protein candidates in which ACBD6, BASP1 and MARCKS had already been confirmed directly interacting with NMT1^19–21^. Pathway enrichment analysis of the results showed that genes involved in mRNA metabolic process, translation initiation and ER localization were significantly enriched (Fig. 3a). This might suggest knocking down NMT1 would abrogate proper protein translation and processing in ER, and caused intracellular degraded protein increase, resulting in ER stress. And we found the level of poly-ubiquitinated proteins increased after NMT1 knockdown (Fig. 3b). Previous reports have demonstrated that aggregation of ubiquitinated proteins would lead to dysfunction in many cellular pathways and intrinsic stress condition like ER stress and oxidative stress^22^. And NMT1 knockdown significantly up-regulated ROS level in breast cancer cells (Fig. 3c and S3A), which clearly indicated NMT1 knockdown in breast cancer could also contribute to oxidative stress.

To explore the function of elevated ROS in NMT1 knockdown breast cancer, we utilized an antioxidant N-acetyl cysteine (NAC) to neutralize ROS. Co-treatment with NAC fully reversed the NMT1 knockdown-induced increase in ROS (Fig. 3d and S3B). NAC treatment also reversed the inhibition effect of NMT1 knockdown on ALDH positive cells population and mammosphere formation (Figs. 3e,f and S3C, S3D). Moreover, NAC promoted the ability of migration and invasion in NMT1 knockdown cells (Figs. 3h, i and S3F, S3G). Most importantly, the ability of cancer cell proliferation was significantly rescued after NAC treatment (Fig. 3G and S3E). Taken together, these results indicated NMT1 inhibition could induce oxidative stress to modulate breast cancer initiation and progression.

Then we determined whether oxidative stress had an influence on NMT1 expression. The protein level but not mRNA level of NMT1 was remarkably improved after NAC treatment, indicating ROS might regulate NMT1 expression in a post-translational manner to create a positive feedback loop (Figs. 3j, k and S3H). To testify this hypothesis, we applied H_2_O_2_, a major source of ROS, to treat SUM149 and MDA-MB-231. ROS could decrease NMT1 protein expression in a dose dependent manner (Fig. 3l and S3I). These data elucidated that excessive ROS hinders breast cancer progression through targeting NMT1 and NMT1 inhibition conversely promoted oxidative stress. As shown in Fig. 3k, NAC treatment slightly elevated NMT1 expression and alleviated ER stress a bit in NMT1 knockdown cells. Moreover, H_2_O_2_ treatment significantly up-regulated ER stress markers expression, indicating ROS positively regulated ER stress (Fig. 3m). Figure 3n and S3J showed that PERK knockdown would partially minimize ROS increase in NMT1 knockdown breast cancer cells, indicating ER stress was one of the ways for producing ROS in these cells.

### JNK pathway plays a key role in breast cancer progression mediated by NMT1 knockdown

**Fig. 4:**
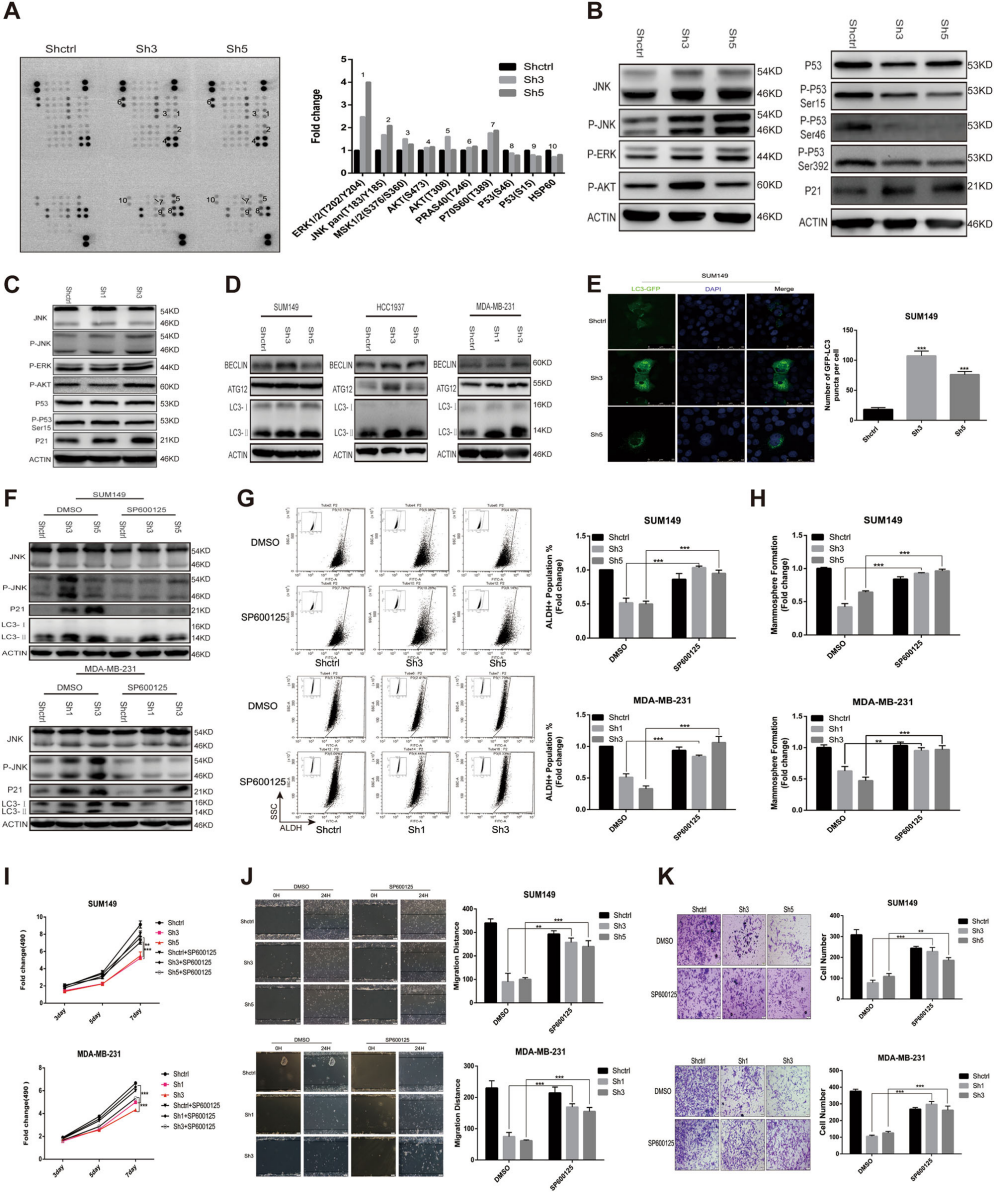
JNK pathway plays a key role in breast cancer progression mediated by NMT1 knockdown. **a** The Human Phospho-Kinase Array was used to detect multiple phosphorylated kinases in Shctrl and ShNMT1-infected SUM149 cells. Template showing the location of kinase antibodies spotted onto the Human Phospho-Kinase Array kit and relevant kinases were indicated by numbers (left). Quantification of mean spot pixel densities of the indicated kinases (right). **b** The indicated kinases were detected in Shctrl and ShNMT1-infected SUM149 cells by western blot. **c** The indicated kinases were detected in Shctrl and ShNMT1-infected MDA-MB-231 cells by western blot. **d** Autophagy related protein expression was determined by western blot in Shctrl and ShNMT1-infected SUM149, MDA-MB-231 and HCC1937 cells. **e** Representative images showing the formation of GFP-LC3 puncta in Shctrl and ShNMT1-infected SUM149 cells (left). GFP-LC3 puncta per cell was quantified (right). **f** Shctrl and ShNMT1-infected SUM149 and MDA-MB-231 cells were treated with SP600125 (20 uM) or same volume of DMSO for 48 h. The expression of JNK, LC3 and p21 were then detected by western blot. **g** Representative images of ALDH-positive cells in the cells from **f** (left). ALDH-positive cells were quantified (right). **h** Quantification of Mammosphere formation from the cells in **f**. **i** MTT assay was used to measure the proliferation of cells in **f**. **j**, **k** Wound healing assay (**j**) and Transwell assay (**k**) were used to measure migration and invasion ability of the cells in **f** as described in methods. Data represent the mean ± SD of 3 independent experiments where *P < 0.05, **P < 0.01 and ***P < 0.001

To gain further insight into the signaling pathways involved in the NMT1 knockdown mediated ER stress and oxidative stress-dependent regulation, we then performed a phosphokinase array to screen for phosphorylation of approximate 50 proteins in SUM149-NMT1Sh cell lines (Fig. 4a). Activation of ERK or AKT was confirmed in SUM149 (Fig. 4b), but not seen in MDA-MB-231 and HCC1937 after NMT1 knockdown (Fig. 4c and S4A). The p53 protein and its phosphorylated forms were down-regulated upon NMT1 knockdown in SUM149 and MDA-MB-231(Fig. 4b,c), whereas hardly detected in HCC1937. The expression of p53 downstream targets in NMT1 knockdown SUM149 and MDA-MB-231 cells indicated that there might be other pathways involved in this process (Fig. S4B). The results revealed significant elevations in the c-Jun N-terminal kinase (JNK) phosphorylation in SUM149 upon knockdown of NMT1 (Fig. 4b), consistent with data obtained in MDA-MB-231 and HCC1937 (Fig. 4c and S4A), indicating JNK might be one of the key regulating pathways. Knocking down NMT1 significantly increased p21 expression to arrest cell proliferation (Fig. 4b,c and S4A), which could be abolished by inhibiting JNK pathway (Fig. 4f and S4F). JNK pathway regulates several important cellular functions including cell growth, invasion, autophagy and apoptosis^23^. As shown in Fig. S4C, apoptotic cells population didn’t increase by NMT1 knockdown. And autophagy related proteins Beclin and Atg12 were elevated (Fig. 4d). NMT1 knockdown increased level of lipidated LC3 in breast cancer (Fig. 4d). Transfection of NMT1 knockdown cells with GFP-LC3 plasmid resulted in an increased abundance of GFP-LC3 puncta (Fig. 4e and S4D). Pharmacological inhibition of JNK pathway totally abrogated the accumulation of the lipidated LC3-II form and LC3 puncta in NMT1 knockdown breast cancer cells (Fig. 4f and S4E). Thus, activation of JNK pathway mediated autophagy process in this situation.

**Fig. 5:**
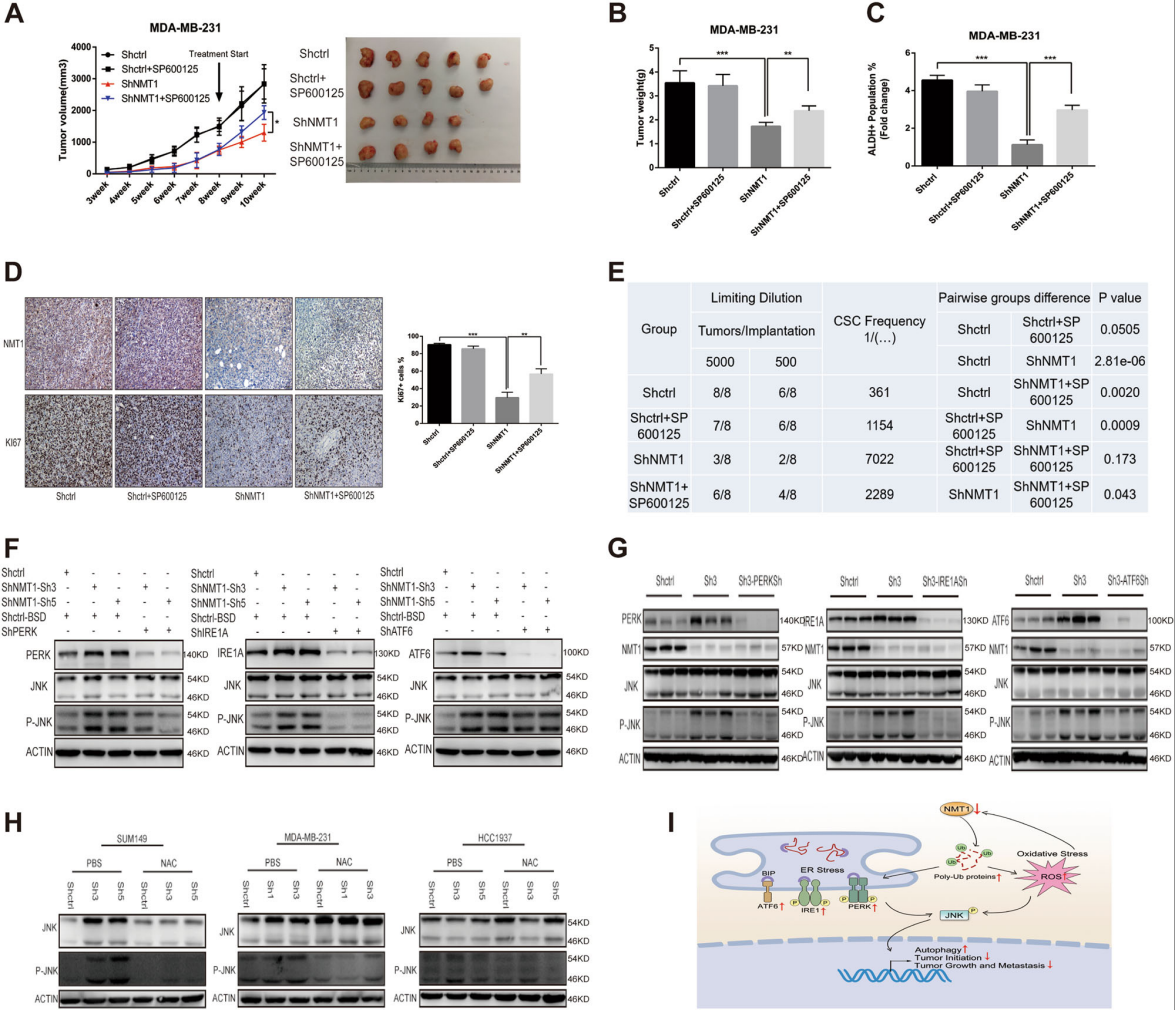
Interplay of oxidative stress, ER stress, and JNK pathway in NMT1 knockdown breast cancer. **a** For each group, 1 million MDA-MB-231 cells were implanted into the mammary gland fat pads of 3-week-old to 4-week-old female nude mice and tumor size was monitored weekly (left).SP600125 treatment (30 mg/kg/day) started from indicated time and lasted for two weeks. The tumor image was shown on the right. **b** Tumor weight from A. **c** Tumors from A were collected and cells were isolated from each tumor. ALDH was accessed by the ALDEFLUOR assay on viable dissociated cells. **d** The representative images for NMT1 and Ki67 IHC staining of tumors from A (left). Ki67 positive cells were counted. **e** Extreme limiting dilution analysis for the four groups in A was calculated on the website http://bioinf.wehi.edu.au/software/elda/. **f** ER stress related genes were knocked down via lentiviral infection in Shctrl and ShNMT1-infected SUM149 cells. Then JNK was detected by western blot. **g** Tumors from Fig. 2-O were collected and cells were isolated from each tumor. Then the cells were lysated and underwent western blot to detect ER stress related proteins, NMT1 and JNK. **h** SUM149, MDA-MB-231 and HCC1937 cells were treated with NAC (10 mM) or same volume of PBS for 48 h. Then the expression of JNK was detected by western blot. **i** The schematic illustration of NMT1 knockdown effects on breast cancer. Data represent the mean ± SD of 3 independent experiments where *P < 0.05, **P < 0.01 and ***P < 0.001

Given the observation that JNK pathway was activated, we needed to elucidate the importance of JNK pathway in breast cancer after NMT1 knockdown. SP600125, a broad-spectrum JNK inhibitor, was utilized to inhibit phosphorylation of JNK of which the effect was confirmed (Fig. 4f). SP600125 significantly abrogated NMT1 knockdown mediated downregulation of ALDH-positive population and mammosphere formation (Figs. 4g,h and S4G, S4H). Meanwhile, inhibition of JNK pathway partially abolished the suppressed effect of NMT1 knockdown on cell proliferation (Fig. 4i and S4I), migration (Fig. 4j and S4J) and invasion (Fig. 4k and S4K). Furthermore, nude mice were injected with MDA-MB-231-NMT1sh cells and then treated with SP600125 for two weeks after tumors reached to a certain size. As shown in Fig. 5a,b, tumors in NMT1 knockdown group treated with SP600125 were larger and heavier than knockdown group itself. And SP600125 remarkably blunted the effect of NMT1 knockdown on ALDH positive population and Ki67 positive population in vivo (Fig. 5c,d). But, the effect of NMT1 knockdown on tumor invasion was not rescued might due to that the treatment time was not enough (Fig. S5A). Next, we determined the ability of serial dilutions of cells obtained from these primary tumors to form tumors in secondary nude mice. NMT1 knockdown decreased the CSC frequency supporting that tumor initiation ability was abrogated by NMT1 inhibition. SP600125 treatment significantly rescued this effect (Fig. 5e). Together, these results indicated JNK pathway activation, caused by NMT1 knockdown, was responsible for NMT1 knockdown mediated breast cancer progression delay both in vitro and in vivo.

### Interplay of oxidative stress, ER stress, and JNK pathway in NMT1 knockdown breast cancer

As mentioned above, JNK pathway played a key role in NMT1 knockdown breast cancer. And we wanted to identify the interplay between JNK and those two intercellular stresses. Our study has demonstrated that knocking down PERK and IRE1A respectively abolished the JNK pathway activation in SUM149-NMT1sh cells (Fig. 5f). Inhibition of PERK or IRE1A could abrogate elevated JNK phosphorylation level in MDA-MB-231-NMT1sh xenografts (Fig. 5g). Whereas inhibition of JNK phosphorylation had no significant effect on the expression of ER stress markers (Fig. S5C). We here found that inhibition of ROS production via NAC significantly restrained JNK pathway activation in NMT1 knockdown breast cancer cell lines (Fig. 5h). Meanwhile, inhibition of JNK phosphorylation slightly reduced the amount of ROS in breast cancer (Fig. 5d), indicating the dual-interplay between JNK and ROS existed under this circumstance. To be noticed, JNK inhibition did not affect NMT1 expression (Fig. S5B). The schematic illustration for underlying mechanisms of how NMT1 knockdown regulating breast cancer initiation, growth and metastasis was illustrated in Fig. 5i.

## Discussion

In this study, we demonstrate that NMT1 is capable of modulating breast cancer cell initiation and promoting cell proliferation and invasion through intracellular stress induced JNK pathway activation both in vitro and in vivo. NMT1 has been reported to promote tumor progression partially due to its myristoylation to some famous oncoproteins, like SFK family kinases^24^. Previous reports demonstrated that Src contributed to JNK activation^25^. However, we did not see any significant changes in the phosphorylated levels of SFK family kinases via genetic inhibition of NMT1 in breast cancer cells. Instead, we found that JNK pathway was activated in NMT1 knockdown breast cancer cells and xenograft tumors. Unexpectedly, specific NMT1 interacting protein associated with JNK pathway could not be identified and overexpression of NMT1 did not affect any significant changes in phenotypes of breast cancer cells, such as cell proliferation and invasion. These results indicated that a simple NMT1–substrate complex might not explain the changes in NMT1 inhibition to breast cancer. MS results showed that NMT1 participated in lots of biological process like mRNA metabolic, translation initiation and ER localization. Consistent with these results, pharmacological inhibition of myristoylation by utilizing a NMT inhibitor named “Compound 1” induced cell cycle arrest and ER stress, leading to apoptosis in cancer cells^12^. Since the “Compound 1” was not specifically aimed to NMT1 and pharmacological inhibition did not last very long time, we did experiments in our genetic NMT1 inhibiting breast cancer model. And we found NMT1 knockdown indeed trigger ER stress but not apoptosis in our system. Based on these findings, we hypothesized that targeting NMT1 would disrupt lots of protein function, and location, and abrogate normally biological process, causing great damage to breast cancer cellular homeostasis.

Tumor cells must evolve by adapting to lots of stress conditions to achieve intercellular homeostasis, ultimately driving tumor progression. Manipulation of this balance might provide therapeutic approaches to eliminate cancer cells, especially for aggressive malignancy, such as triple-negative breast cancers. In our study, NMT1 knockdown by ShRNA significantly collectively induced three parallel ER stress signaling pathways mediated by three main effectors: PERK, IRE1A, and ATF6. Previous reports have demonstrated that ER stress was essential for cancer cell initiation, proliferation, invasion and apoptosis^26–28^. But the role of ER stress in cancer cell proliferation has been controversial. For example, PERK-EIF2α axis caused growth arrest in vitro and suppressed tumor growth in vivo in established tumors^29,30^, whereas PERK-NRF2 axis promoted cancer cell proliferation in breast cancer mice^31^. Collectively, these observations illustrated the complexity and dynamic of ER stress. The genetic inhibition of NMT1 triggered ER stress was persisted and prolonged in breast cancer cells, which had promising effects on many other biological aspects. It is reported that PERK mediated ER-mitochondrial crosstalk and prolonged PERK activation increased production of ROS^32^. It’s also well established that IRE1A could recruit TRAF2 during ER stress and therefore activated JNK and NF-ΚB signaling^33,34^. In our study, not only IRE1A but also PERK could lead to activation of JNK pathway. And consistent with previous reports, elevated ROS production partially depend on PERK in NMT1 knockdown cells, which might explain the reason why PERK was associated with JNK activation since ROS triggered oxidative stress was believed to activate JNK pathway. Conversely, abundant ROS could cause severe ER stress, which was reported previously^32^ and confirmed in our experiments.

Cancer cells rely on the signaling capabilities of ROS for cell invasion, proliferation, and survival. Importantly, if ROS levels are too high, ROS can promote anti-tumorigenic signaling and trigger oxidative stress-induced cancer cell senescence and cell death to inhibit tumor growth and metastasis^35^. ROS was produced at higher level in NMT1 knockdown breast cancer cells than shctrl. There were two possible mechanisms of ROS regulating this process: one way was JNK pathway activation, the other was the feedback loop of JNK-independent ROS down-regulating NMT1. It was well established that the role of JNK pathway in cancer was dependent on the stimulation type, strength and tissue specificity^36^. Whereas transient JNK activation was shown to promote cell survival, prolonged JNK activation mediated anti-tumorigenic effect^37^. Previous reports demonstrated that JNK signaling regulated lots of ATG genes to promote autophagy^38^. Our study suggested that JNK activation promoted not apoptosis but autophagy and up-regulated downstream target p21 to prevent breast cancer progression after NMT1 knockdown. Besides, inhibition of JNK tended to slightly but significantly decrease ROS production. In other way, we determined that H_2_O_2_ could regulate NMT1 expression in a dose dependent manner. And inhibition of ROS via NAC remarkably elevated NMT1 protein level. These results demonstrated that oxidative stress could cause NMT1 inhibition and then NMT1 expression reduction promoted ROS production through degraded proteins accumulation, ER stress and JNK activation, which formed a tight feedback loop. These results provided a new mechanism of how oxidative stress abrogated breast tumor progression.

Recently, some NMT inhibitors including “Compound 1” and B13 have been developed as anti-tumor agents^11,12^. But the mechanisms of targeting NMT1 to suppress cancer progression were not clearly illustrated yet. We have demonstrated that prolonged inhibition of NMT1 could cause poly-ubiquitinated proteins increase, trigger ER stress and oxidative stress, and result in JNK abnormal activation in breast cancer. Our study should allow us to better understand the mechanisms and factors involved in this process that may help to improve diagnostic and therapeutic approaches to breast cancer.

## Electronic supplementary material

Table S1

Table S2

Supplementary figure legends

Figure S1

Figure S2

Figure S3

Figure S4

Figure S5
