# Supplementary material for: NMT1 inhibition modulates breast cancer progression through stress-triggered JNK pathway
Source: Cell Death Dis. 2018 Nov 16;9(12):1143. doi: 10.1038/s41419-018-1201-x (PMC6240078; doi:10.1038/s41419-018-1201-x)
Supplement: Supplementary file 3 — Supplementary figure legends [file 41419_2018_1201_MOESM3_ESM.docx]

**Figure S1**．**NMT1 knockdown inhibits breast cancer progression**

A. SUM149 and MDA-MB-231 cells were transduced with the pTRIPZ-miR100 lentivirus and selected with puromycin for 7 days.Cells were treated with (MiR100) or without (CTRL) tetracycline (doxycycline; DOX). Total RNA was isolated and miR100 expression level were measured by qRT-PCR (left). The expression of NMT1 was detected in the cells above by qRT-PCR and western blot (right). B. The expressions of NMT1 was detected by western blot across different breast cell lines, including: 1) normal mammary gland cell lines, MCF10A and HBL100; 2) luminal breast cancer cell lines, MCF7 and T47D (ER+PR-HER2-); 3) HER2+breast cancer cell lines (ER-PR-HER2+) containing SKBR3, BT474; 4) Basal-like/TNBC (ER-PR-HER2-) breast cancer cell lines, such as MDA-MB-468,HCC1937, SUM149, SUM159 and MDA-MB-231.C. NMT1 was knocked down via Shctrl and ShNMT1 lentiviral infection. NMT1 expression was detected by qRT-PCR and western blot in HCC1937 and T47D.D. ALDEFLUOR assay was performed in the Shctrl and ShNMT1-infected HCC1937 and T47D cells. E. Quantification of ALDH-positive cells in C. F. Mammosphere formation assay in Shctrl and ShNMT1-infected HCC1937 and T47D cells. Number of mammosphere was calculated (n > 3). G-H. MTT assay (G) and colony formation assay (H) were used to measure cell proliferation as described in methods. I. Cell cycle distribution analyzed by FACS in Shctrl and ShNMT1-infected HCC1937 and T47D cells. J. Cell cycle related proteins were detected by western blot in Shctrl and ShNMT1-infected HCC1937 and T47D cells. K-L. Wound healing assay (K) and Transwell assay (L) were used to measure cell migration and invasion ability as described in methods. M. The representative images for NMT1 IHC staining in tumors form Figure 1-L. N. NMT1 expression in tumors from Figure 1-L was measured by western blot. Data represent the mean ±SD of 3 independent experiments where **P* < 0.05, ***P* < 0.01 and ****P* < 0.001.

**Figure S2. NMT1 knockdown triggers ER stress**

A. ER stress related genes expression was determined by qRT-PCR in Shctrl and ShNMT1-infected HCC1937 and T47D cells. B. ER stress related protein expression was detected by western blot in cells from A.C. Shctrl and ShNMT1-infected HCC1937 and T47D cells were treated with 4-PBA (2 um) or same volume of DMSO for 48h. ER stress markers were then determined by Western blot. D. Quantification of ALDH-positive cells in 4-PBA or DMSO treated Shctrl and ShNMT1-infected HCC1937 cells. E. Quantification of Mammosphere formation in cells from D.F. MTT assay for cells in D as described in methods. G-H. Wound healing assay (G) and Transwell assay (H) was used to measure the migration and invasion ability of cells in D as described in methods I. ER stress related genes were knocked down via lentiviral infection in Shctrl and ShNMT1-infected HCC1937 cells. The expression of PERK, IRE1A and ATF6 was detected by western blot. J. Quantification of ALDEFLUOR-positive cells from the cells in I.K. Quantification of Mammosphere formation from the cells in H.L. MTT assay was used to measure the proliferation of cells in H.M-N. Wound healing assay (M) and Transwell assay (N) were used to measure the migration and invasion ability of cells in H as described in methods. O. SUM149 was treated with BFA (2uM) for 48h.Then ER stress related proteins and NMT1 were detected by western blot. P. SUM149 was treated with 4-PBA (2uM) for 48h.Then ER stress related proteins and NMT1 were detected by western blot. Q. The representative images for NMT1 IHC staining in tumors from Figure 2O.Data represent the mean ±SD of 3 independent experiments where **P* < 0.05, ***P* < 0.01 and ****P* < 0.001.

**Figure S3. NMT1 knockdown promotes ROS elevation to inhibit breast cancer progression**

A. Representative flow cytometry curves of total intracellular ROS levels (H2DCFDA) in Shctrl and ShNMT1-infected HCC1937 cells (left). Quantification of ROS levels (right).B. Representative flow cytometry curves of total intracellular ROS levels (H2DCFDA) in Shctrl and ShNMT1-infected HCC1937 cells following 48h of exposure to PBS or NAC (10mM) (left). Quantification of ROS levels (right).C. Shctrl and ShNMT1-infected HCC1937 cells were treated with NAC (10mM) or same volume of PBS for 48h. ALDH was accessed by the ALDEFLUOR assay and ALDH-positive cells were calculated. D. Quantification of Mammosphere formation from the cells in C.E. MTT assay was used to measure the proliferation of cells in C.F-G. Wound healing assay (F) and Transwell assay (G) were used to measure the migration and invasion ability of cells in C as described in methods. H. HCC1937 cells were treated with NAC (10mM) or same volume of PBS for 48h. Then the expression of NMT1 was detected by qPCR. I. SUM149 and MDA-MB-231 cells were treated with indicated concentration of H_2_O_2_ for 48h. The mRNA level of NMT1 was detected by qRT-PCR.J. ER stress related genes were knocked down via lentiviral infection in Shctrl and ShNMT1-infected HCC1937 cells. Quantification of total intracellular ROS levels (H2DCFDA) in these cells. Data represent the mean ±SD of 3 independent experiments where **P* < 0.05, ***P* < 0.01 and ****P* < 0.001.

**Figure S4. NMT1 knockdown stimulates JNK pathway to trigger autophagy**

A. Shctrl and ShNMT1-infected HCC1937 cells were treated with SP600125 (20uM) or same volume of DMSO for 48h. The expression of JNK, LC3 and p21 were then detected by western blot. B. Representative flow cytometry images of apoptotic cells in Shctrl and ShNMT1-infected SUM149 and MDA-MB-231 cells. C. Representative images showing the formation of GFP-LC3 puncta in Shctrl and ShNMT1-infected MDA-MB-231 cells (left). GFP-LC3 puncta per cell were quantified (right).D. Representative images showing the formation of GFP-LC3 puncta in Shctrl and ShNMT1-infected HCC1937 cells (left). GFP-LC3 puncta per cell were quantified (right).E. Representative images showing GFP-LC3 puncta in Shctrl and ShNMT1-infected HCC1937 cells following the treatment of SP600125 (20uM) or same volume of DMSO for 48h (left). GFP-LC3 puncta per cell were quantified (right).F. Shctrl and ShNMT1-infected HCC1937 cells were treated with SP600125 (20uM) or same volume of DMSO for 48h. The expression of JNK, LC3 and p21 was then detected by western blot. G. Representative images of ALDH-positive cells in the cells from F (left). ALDH-positive cells were quantified (right).H. Quantification of Mammosphere formation from the cells in F.I. MTT assay was used to measure the proliferation of cells in F.J-K. Wound healing assay (J) and Transwell assay (K) were used to measure the migration and invasion ability of cells in F as described in methods. Data represent the mean ±SD of 3 independent experiments where **P* < 0.05, ***P* < 0.01 and ****P* < 0.001.

**Figure S5. The correlation of JNK pathway with ER stress and oxidative stress.**

A. The representative images for HE staining of lung sections from Figure 5A (left). The numbers of metastatic lesions per lung section were counted (right). B. SUM149, MDA-MB-231 and HCC1937 were treated with SP600125 (20 uM) or DMSO for 48h.Then JNK and NMT1 were detected by western blot. C. SUM149 was treated with SP600125 (20 uM) or DMSO for 48h. Then ER stress related proteins were detected by western blot. D. SUM149 and MDA-MB-231 were treated with SP600125 (20 uM) or DMSO for 48h. Representative flow cytometry curves of total intracellular ROS levels (H2DCFDA) in these cells (left). Quantification of ROS levels (right). Data represent the mean ±SD of 3 independent experiments where **P* < 0.05, ***P* < 0.01 and ****P* < 0.001.
