# Supplementary figures and images for: NMT1 inhibition modulates breast cancer progression through stress-triggered JNK pathway
Source: Cell Death Dis. 2018 Nov 16;9(12):1143. doi: 10.1038/s41419-018-1201-x (PMC6240078; doi:10.1038/s41419-018-1201-x)

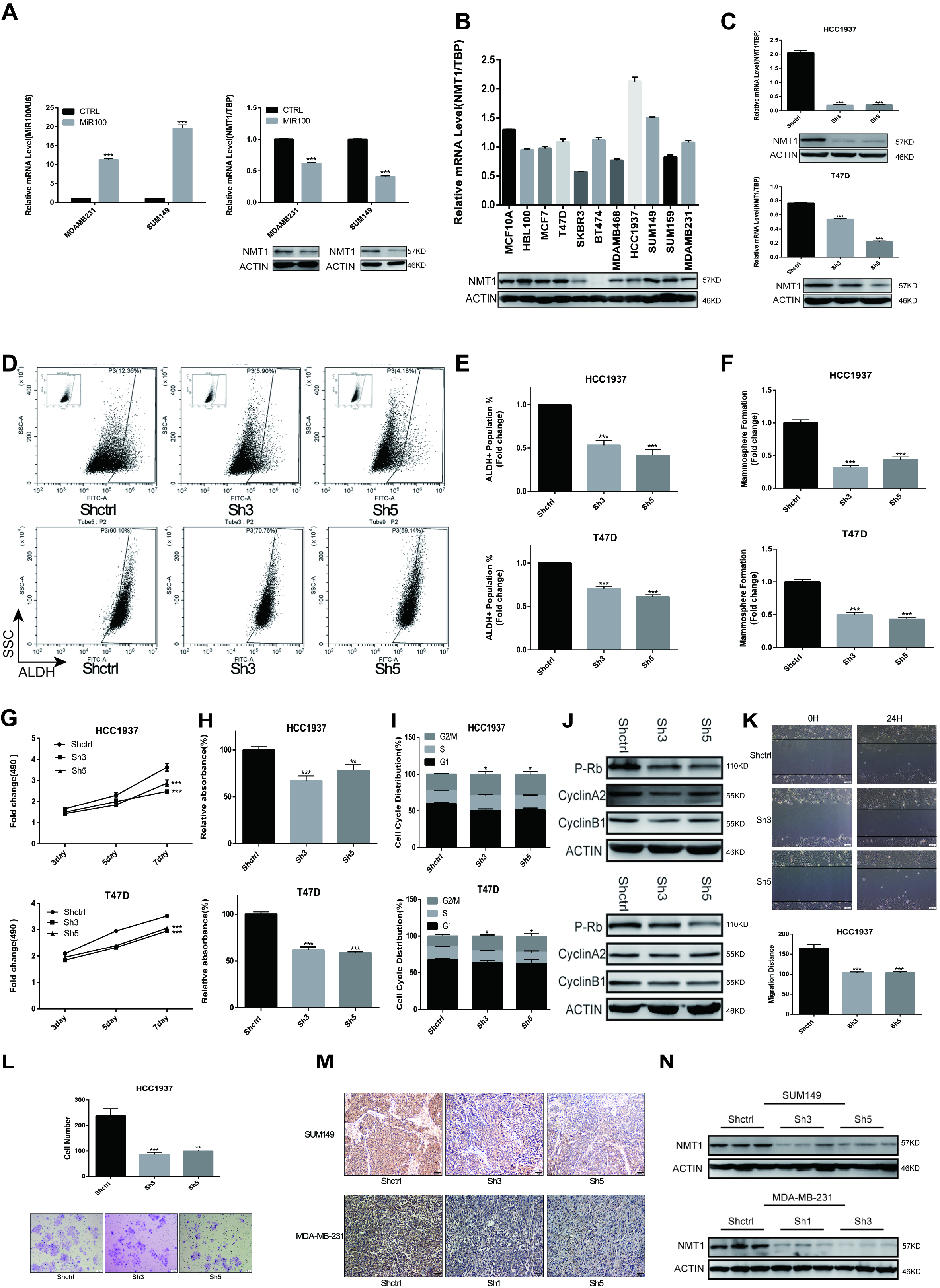

Supplement: Supplementary file 4 — Figure S1 [file 41419_2018_1201_MOESM4_ESM.tif]

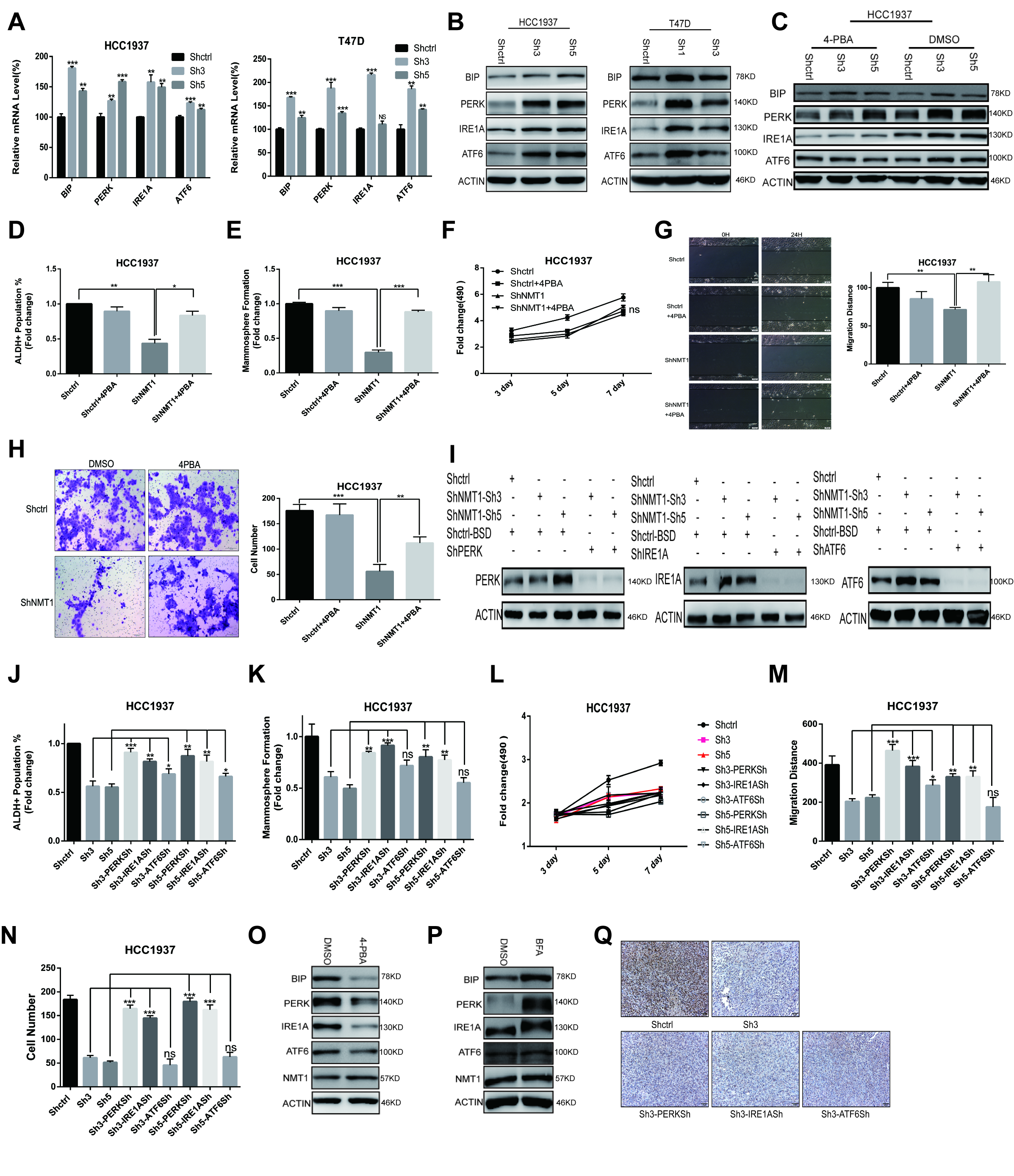

Supplement: Supplementary file 5 — Figure S2 [file 41419_2018_1201_MOESM5_ESM.tif]

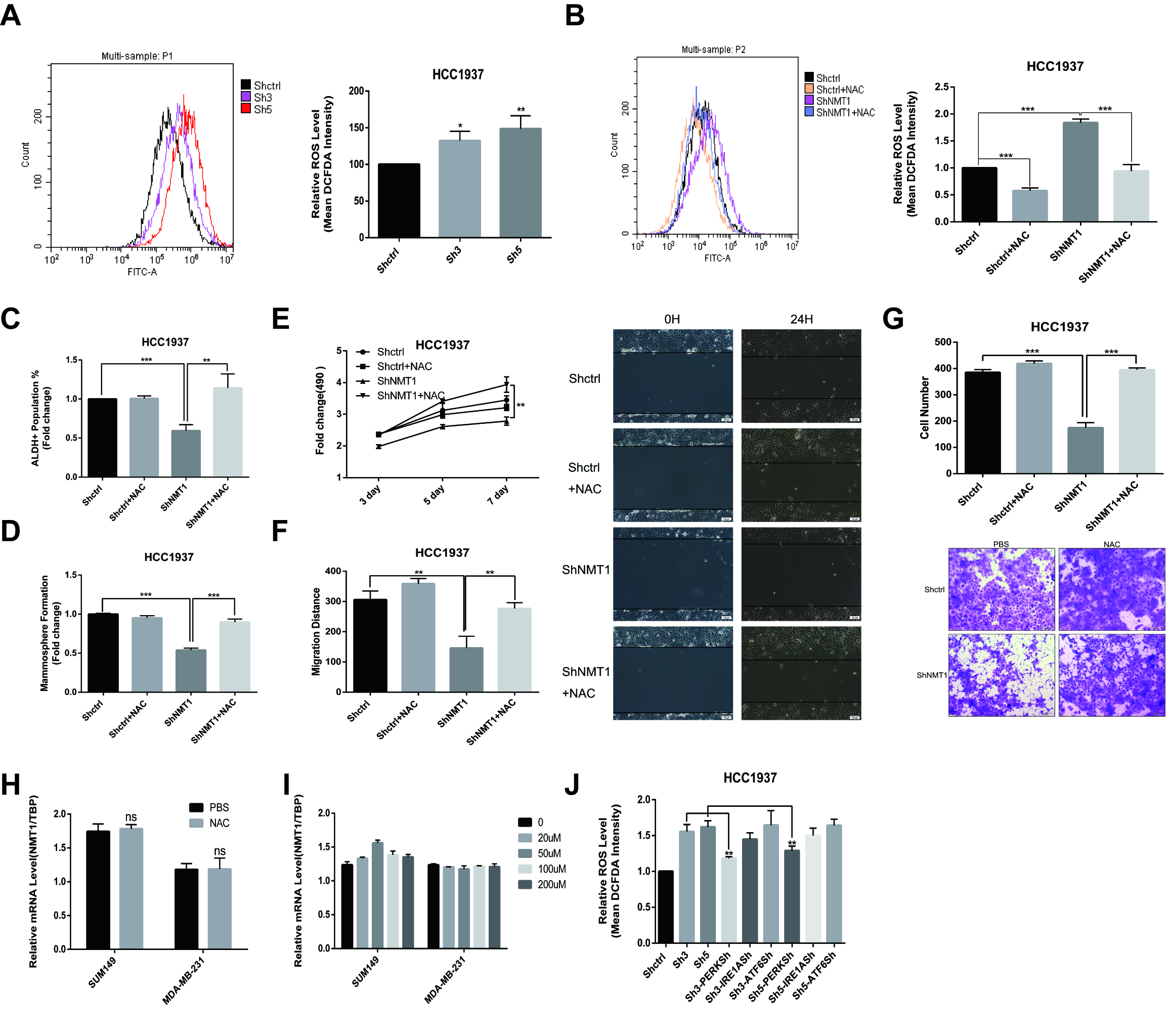

Supplement: Supplementary file 6 — Figure S3 [file 41419_2018_1201_MOESM6_ESM.tif]

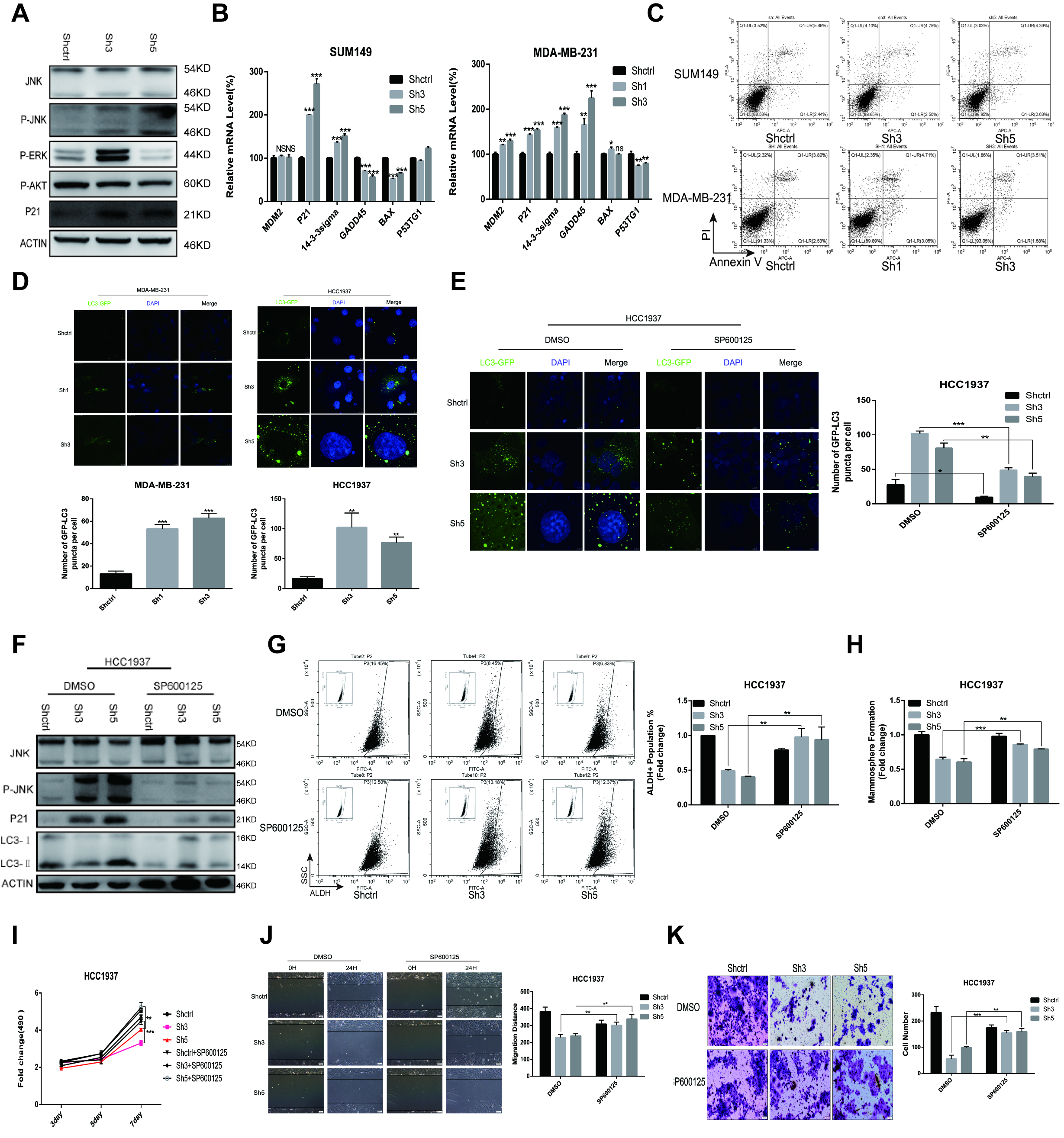

Supplement: Supplementary file 7 — Figure S4 [file 41419_2018_1201_MOESM7_ESM.tif]

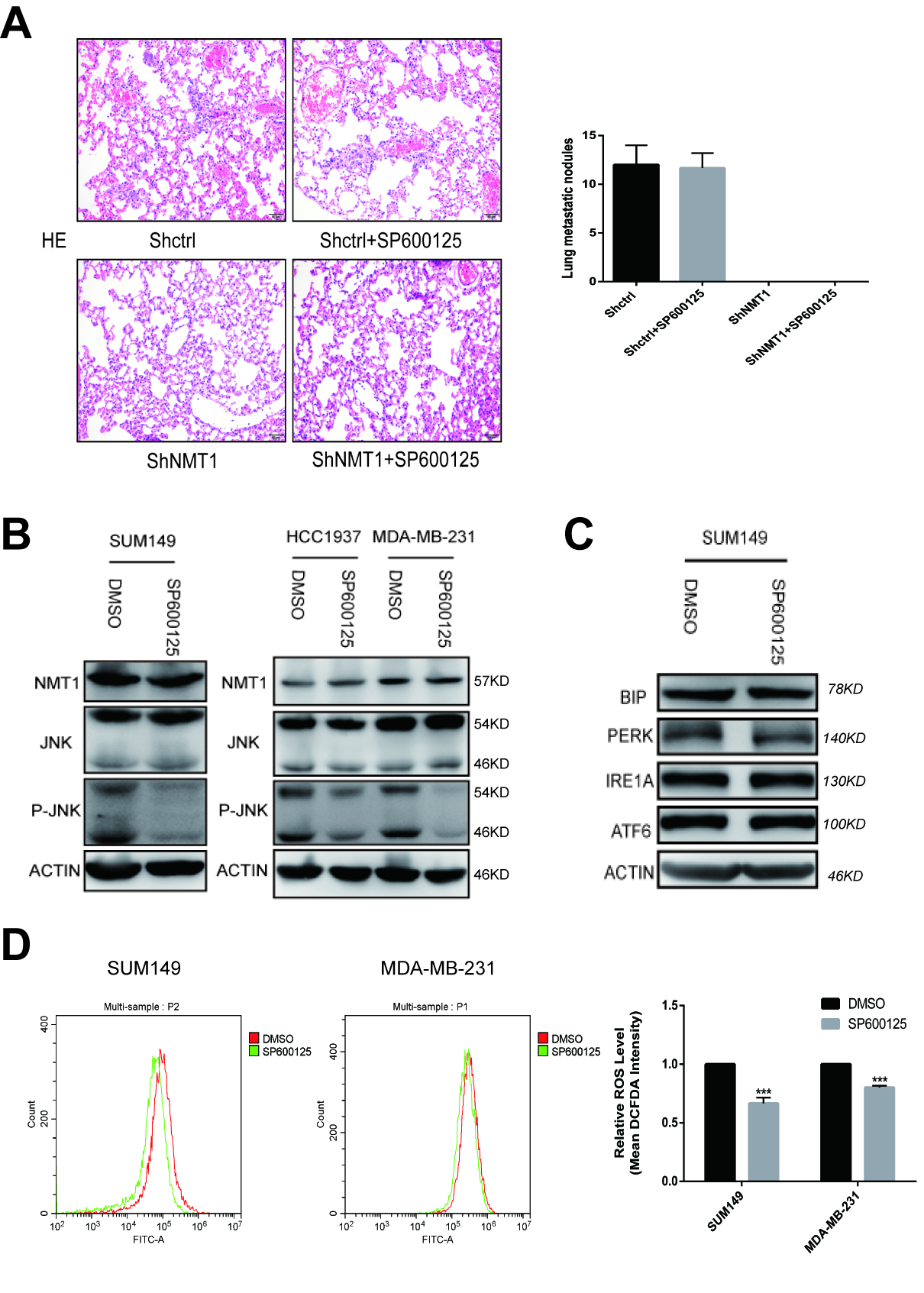

Supplement: Supplementary file 8 — Figure S5 [file 41419_2018_1201_MOESM8_ESM.tif]
